# Activation levels of plausible alternatives in conversational negation

**DOI:** 10.3758/s13421-023-01434-2

**Published:** 2023-07-05

**Authors:** Francesca Capuano, Theresa Sorg, Barbara Kaup

**Affiliations:** Department of Psychology, University of Tübingen, Schleichstraße 4, 72076 Tübingen, Germany

**Keywords:** Negation, Pragmatics, Alternatives, Activation levels, Priming

## Abstract

Negation is often used to contradict or correct (e.g. *There is no dog here.*). While rejecting some state of affairs that is presumed to hold for the recipient (e.g. *There is a dog here.*), the speaker might implicitly suggest a set of plausible alternatives (e.g. *There is a wolf instead.*). Prior work indicates that alternatives are highly relevant to the comprehension of sentences involving focus: in priming studies, listeners infer plausible alternatives to focused items even when they are not contextually available. So far it is unclear whether negation similarly activates an automatic search for plausible alternatives. The current study was designed to investigate this question, by looking at the activation levels of nouns after negative and affirmative sentences. In a series of priming experiments, subjects were presented with negative and affirmative sentences (e.g. *There is an/no apple.*), followed by a lexical decision task with targets including plausible alternatives (e.g. *pear*), as well as semantically related but implausible alternatives (e.g. *seed*). An interaction of Sentence Polarity and Prime-Target Relation was expected, with negation facilitating responses to plausible alternatives. Results of the first experiment were numerically in line with the hypothesis but the interaction just missed significance level. A post hoc analysis revealed the expected significant interaction. Possible roles of sentential context and goodness of alternatives are discussed. A further experiment confirms that the goodness of alternatives is in fact critical in modulating the effect.

It has been long pointed out that one of the primary functions of negation in natural language is to contradict or correct (Clark and Clark, 1977; Givón, 1978; Wason, 1965; Kaup, 2009; Horn, 1989). The use of negation often presupposes the understanding of the speaker that the listener might erroneously believe the negated state of affairs to hold. Schindele et al. (2008) showed that Theory of Mind processes, i.e. the ability to put oneself in the mental state of the other person, are indeed necessary to understand the pragmatic aspects of negation. In Kruszewski et al. (2016) speakers judged a sentence like *It’s not a dog, it’s a wolf* as more plausible than a sentence like *It’s not a dog, it’s a screwdriver*. More specifically, they show a correlation to exist between the semantic similarity of the negated entity (*dog*) and its alternatives (*wolf* vs. *screwdriver*), and the plausibility judgments of the sentences: the more similar the two entities (e.g. *dog* and *wolf*), the more plausible was the sentence rated. Possibly, higher similarity corresponds to higher confusability between the two entities, therefore licensing the assumption of the speaker on the false presupposition held by the listener.

Capuano et al. (2021) confirmed that the preference for highly similar alternatives is indeed specific to negation, going beyond a general preference for semantically similar nouns within the same sentence. In a series of cloze tasks, they collected completions to four different minimal sentential contexts, both in the negative and in the affirmative form (e.g. *There is no dog here, but there is ________* vs. *There is a dog here, and there is a ________ there.*)). In three out of four contexts, completions to the negative sentences appeared to be significantly more similar to the first-mentioned entity than in the case of the affirmative sentences, indeed confirming this to be a peculiarity of negation. In this study subjects were explicitly prompted for an alternative. To our knowledge, it has not been investigated whether negation automatically activates a search for plausible alternatives in case no alternative were explicitly solicited.

Alternatives are key to the successful comprehension of certain types of utterances. Following the work of Rooth (1992) on alternative semantics, focused items evoke alternatives which are relevant to the interpretation of an utterance (e.g. *[Mary]*_*F*_*likes Sue* evokes the set of propositions of the form *x likes Sue*, whereas *Mary likes [Sue]*_*F*_ evokes the set of propositions of the form *Mary likes x*). Recent priming studies have tapped into the psychological reality of this semantic theory, showing that listeners do infer alternatives to focused items, even when (Yan and Calhoun 2019; Braun and Tagliapietra 2010; Husband and Ferreira 2016; for a more comprehensive overview, see Gotzner and Spalek 2019). Using cross-modal lexical decision tasks, Braun and Tagliapietra (2010) investigated semantic priming of sentences differing in intonation pattern and in the semantic relation between the final word and the target word in Dutch. When an utterance was contrastively accented (e.g. *In****Florida****he photographed a****flamingo***), contextual alternatives (e.g. *pelican*) were activated preferentially compared to a neutral intonation condition, but non-contrastive semantic associates (e.g. *pink*) were not. Husband and Ferreira (2016) explored the time course of the priming effect in English, finding evidence for an initial activation of both contrastive and non-contrastive associates, followed by the selective deactivation of non-contrastive associates in the case of contrastively accented sentences. Yan and Calhoun (2019) shows preferential priming of contextual alternatives in Mandarin Chinese when the focus is realised via prosodic prominence, but not when it is realised only syntactically.

Alternatives might be equally relevant to the comprehension of negation. In fact, whereas negation comprehension has been translated psychologically to the general activation of inhibitory mechanisms (de Vega et al., 2016; Beltrán et al., 2019; Liu et al., 2020; Beltrán et al., 2021), the corrective function often linguistically attributed to this construction could find a psychological correlate in the selection of plausible alternatives (either by enhanced activation of plausible alternatives or by inhibition of implausible ones), similarly to what happens with contrastive focus. In that case, we might expect plausible alternatives to be activated preferentially after negative rather than after affirmative statements. This would be in line with existing evidence on activation levels after negation processing. Orenes et al. (2022, Experiment 1), using the priming methodology, found that negation does not decrease the activation level of the mental representation of the negated entity. For example, they found that there was no significant difference in reading time between the target assertions *There were roses and there were lilies* after reading a negated conjunction (*It is not the case that there are roses and there are lilies*) and after reading an affirmative conditional (*If there are roses, then there are lilies*). MacDonald and Just (1989), whilst observing that negation did decrease the activation level of the negated entity (inhibition), on the other hand found that concepts related to the negated entity were not significantly less active than in the case of affirmation. Therefore, negation might (or not) inhibit the activation of the negated concept but no evidence is found for the inhibition of its associates. Crucially, MacDonald and Just made no differentiation between associates that are also plausible alternatives with respect to the negated entity (e.g. *wolf* in relation to *dog*), and associates that are not (e.g. *bone* in relation to *dog*). Therefore, their results do not preclude the possibility of a different pattern for plausible vs. implausible alternatives, and specifically a facilitation of the first with respect to the latter.

Additionally, there is evidence that - whether mediated by the representation of the negated state of affairs (two-simulation hypothesis, see Kaup and Zwaan (2003); Kaup et al. (2007, 2005)) or not (e.g. Mayo et al. 2004) - speakers eventually reach a representation of the actual state of affairs as a result of processing negative statements. Kaup et al. (2006) presented subjects with sentences involving contradictory predicates (e.g. *The door is (not) closed/open.*). The subjects were then presented with a picture and asked to name aloud the entity depicted. The entity was varied according to whether it corresponded to the described or to the negated state of affairs. According to the sentence *The door is closed*, a closed door would correspond to the actual state and an open door would not. On the other hand, according to the sentence *The door is not closed*, an open door would correspond to the actual state and a closed door would not. In order to investigate the temporal characteristics of the representational process, the delay at which the image was presented was varied. One half of the subjects was presented with the image after 750 ms, the other half only after 1500 ms. With a delay of 750 ms, an effect of agreement was found in relation to the actual state in affirmative sentences (e.g. a closed door after the sentence *The door is closed.*), but not in negative sentences (e.g. an open door after the sentence *The door is not closed.*). With a delay of 1500 ms, this effect was also present for negative sentences - the subjects had thus mentally represented the actual state at this point in time, while at an earlier point in time the state to be negated was represented.

A sentence like *The door is not closed* might likely be uttered assuming that the person being addressed thought that the door was closed, and the speaker wanted to make them aware that this was not the case. In this example, the person addressed can easily infer the alternative state (an open door). In fact, *open* and *closed* are contradictory predicates, representing the only two possible states. The actual state of affairs (the alternative) is therefore confined to one possibility here. However, if one looks at the previously mentioned sentence *This is not a dog*, it is harder to determine what the actual state of affairs might be. Seen from the perspective of set theory, any member of the complement set of *dog* would be consistent with *not a dog*. As we have seen though, some entities are more likely alternatives than others: in the case of words that don’t relate to a direct opposite, negation acts a graded similarity function that produces a probability distribution over a restricted set of alternatives (Kruszewski et al., 2016). Given this uncertainty, it is unclear whether listeners still activate likely alternatives in their mental representation.

Based on these considerations, the present study will investigate whether negation leads to the activation of plausible alternatives. For this purpose, we constructed affirmative and negative prime sentences, each involving a concrete entity (e.g. *There is an/no apple.*). The presentation of the sentences was followed by a lexical decision task. Targets could either constitute a plausible alternative (e.g. *pear*) or a semantically related but implausible alternative (e.g. *seed*) with respect to the entity in the prime sentence. Unrelated words were also presented as targets for the sake of a manipulation check: as per literature, we expected both plausible and implausible alternatives to be responded to faster than unrelated words. Non-words were also used as targets to complete the lexical decision task. The experiment was conducted in German. Our main hypothesis was that, assuming negation leads to a search for alternatives, the difference in RTs between negative and affirmative sentences should be smaller in the case of plausible alternatives, compared to semantically related but implausible alternatives. More specifically, an interaction effect of Sentence Polarity and Prime-Target Relation is expected, with negation facilitating responses to plausible alternatives. Secondarily, in line with the literature on negation resulting in increased processing times, a main effect of Polarity can be expected. Nevertheless, no specific prediction is made for the main effect of Polarity within each Prime-Target Relation level: when we say that we expect negation to facilitate responses to plausible alternatives, we do not mean that we necessarily expect negation to display faster reaction times than affirmation to plausible alternatives, because we cannot rule out an underlying main effect of Polarity. Similarly, slower reaction times for implausible alternatives might not signal deactivation in absolute terms because they could be confounded with the slowing effect of negation. What stays informative in light of these considerations is the interaction effect, because any pattern of results displaying a reduced effect of negation in the plausible alternatives condition is consistent with the hypothesis of their *facilitated* activation after negation, although an inhibition of implausible alternatives should equally not be ruled out. In this sense, *facilitation of plausible alternatives* is an umbrella expression for both possibilities.

## Experiment 1

### Participants

Data was collected until 240 usable participants were reached. The sample size was determined based on a previous version of this study which employed slightly different materials that later on turned out to be inadequate. This will be elaborated on in the General Discussion. In total, 294 subjects were tested (201 female, 89 male, 4 diverse) with an age range of 19 to 59 years ($mean=24.52$, $sd=7.60$). Subjects who stated that they were not native German speakers were excluded ($n = 8$) as well as subjects who stated to have already participated in a very similar study (e.g. the previous version of this study) ($n = 27$). The recruitment took place partly via the mail server of the University, and partly via Prolific (Palan and Schitter, 2018). All subjects gave written informed consent.

### Materials

We created affirmative and negative prime sentences of the form *There is [a/an]/[not a/not an] X* (German *Dort ist [ein/e]/[kein/e] X*). Ninety-six common nouns were selected to replace *X*, once in the affirmative and once in the negative form. For each of the 96 nouns (e.g. *apple*), two target words were selected, varying in terms of the relationship they bear with the noun: one plausible alternative (e.g. *pear*) and one semantic associate that is an implausible alternative (e.g. *seed*). Additionally, one semantically unrelated noun (e.g. *brush*) was added for the sake of a sanity check. In fact, both plausible and implausible alternatives should be primed more than an unrelated word. In order to help us construct these items, we ran a cloze task prior to the main study. For this task we chose 100 concrete high frequency nouns (e.g. *apple*). One hundred subjects were instructed to complete sentences such as *This is not an apple, it’s ________* with either just a noun (e.g. *pear*) or an indefinite article plus a noun (e.g. *a pear*). Like the main experiment, the cloze task was run in German. The resulting cloze frequencies helped us construct the majority of the materials (77 out of 96 items) in that, where permitted by the length and frequency match requirements (see below), the plausible alternatives were selected among frequent cloze answers. The remaining items ($n = 19$) were crafted by the authors by intuition. The implausible alternatives were selected among nouns with high similarity that were not good cloze completions. We took care that the implausible alternatives were not cohyponyms, as the post hoc analysis in Capuano et al. (2021) suggests that cohyponyms are particularly good alternatives to negated nouns, at least as far as minimal contexts are concerned.

We controlled for length (number of characters) and frequency (raw counts) between targets across target relations. The mean length for the *plausible alternatives* was 6.43 ($sd=2.41$), 6.17 ($sd= 1.97$) for the *implausible alternatives* and 6.36 ($sd= 1.83$) for the *unrelated* targets. Plausible alternatives did not differ from implausible alternatives ($t(95) = 0.91$, $p =.37$), nor from unrelated targets ($t(95) = 0.20$, $p =.84$). Implausible alternatives and unrelated targets were also paired ($t(95) = -0.69$, $p =.49$).

Target frequency counts were extracted from the deWaC corpus (Baroni et al., 2009) and employed for the matching procedure. The mean frequency for the plausible alternatives was 20906 ($sd=56102$), 24946 ($sd=41120$) for implausible alternatives and 27328 ($sd=95932$) for unrelated targets. All the pairs of conditions were matched: plausible and implausible alternatives ($t(95)=-0.56$, $p=.58$), plausible alternatives and unrelated ($t(95)=-0.57$, $p=.57$), implausible alternatives and unrelated ($t(95)=-0.22$, $p=.83$).

Additionally, cosine similarity scores were calculated for each pair of noun in the sentence (X) and target, employing the LSAfun package with the dewak100k lsa Wordspace (Günther et al., 2015). Plausible alternatives were significantly more similar to the noun ($mean=0.67$, $sd=0.20$) not only with respect to unrelated targets ($mean=0.21$, $sd=0.14$; $t(95)=18.15$, $p<.001$), but also to implausible alternatives ($mean=0.48$, $sd=0.21$; $t(95)=6.22$, $p<.001$). Implausible alternatives were more similar to the noun than unrelated targets ($t(95)=11.61$, $p<.001$). A match between plausible and implausible alternatives could not easily be achieved, as cohyponyms (plausible alternatives) normally tend to score higher on similarity scores than nouns in other semantic relationships (implausible alternatives). Although desirable to achieve an even cleaner design, the match between plausible and implausible alternatives is not needed for the testing of our main hypothesis, since we are testing for the interaction of Polarity and Relation, not for the main effect of Relation.

The two polarity levels of the sentence (*affirmative* vs. *negative*) and the three relation types between the noun in the sentence and the target (*plausible alternative* vs. *implausible alternative* vs. *unrelated*) resulted in six experimental conditions. The conditions were counterbalanced across participants, resulting in a total of six experimental lists with 96 experimental items each. In the experimental material, some words appeared as targets to more than one item. It was taken care that no target would appear more than once in any single list. Additionally, 96 filler sentences were created. These were all in the form of the experimental sentences (e.g. *There is [a/an]/[not a/not an] X*). The target words for the fillers were non-words that were created with the help of the pseudo-word generator Wuggy (Keuleers and Brysbaert, 2010) from the experimental targets of the corresponding list. Each experimental list thus contained 96 experimental sentences (48 affirmative, 48 negative) with 96 target words (32 for each type of relation) and 96 filler sentences with 96 non-words as targets.

In order to ensure that the participants had read the sentences and, above all, had processed the negation, they were prompted to re-type the previously read sentence for 48 of the 192 trials. Half of these were experimental sentences and the other half were filler sentences. Forty usable subjects were collected for each list. The complete collection of items, together with the collected data and analyses scripts of all the experiments presented in this paper can be found at https://osf.io/p5g8u/?view_only=3f40dc06bfa84ac09ce66120c6e71ae4. The experi- ment was programmed using jsPsych (De Leeuw, 2015), a JavaScript library that can be used to create online experiments.

### Procedure

The task of the participants was to read each sentence presented on the screen, then judge whether a target string presented thereafter was either an existing word or a non-word (lexical decision task). At the beginning of each trial, the word *Attention*^1^ appeared in red for 500 ms. A white screen was then shown for 200 ms, followed by the sentence, presented in its entirety (e.g. *There is no apple*). The participants could read the sentence at their own pace, then press the space bar to proceed. According to Kaup et al. (2006), speakers arrive at the factual representation of negative sentences some time between 750 and 1500 ms after sentence processing. We therefore start off with a delay of 1000 ms. After pressing the space bar, a fixation cross was presented for 1000 ms, then the target string appeared (e.g. *pear*). Participants were instructed to react as quickly as possible by pressing either one of two buttons: *k* for words and *d* for non-words. If the target was not responded to within 3500 ms, the message *Too slow! Please react faster!* was displayed and the experiment would proceed to the next trial; otherwise, feedback was provided on the correctness of the lexical decision. After the lexical decision task, subjects were occasionally prompted to re-type the previously read sentence in an input field. The initial instructions were followed by 10 practice trials. Then, the randomized 192 trials from the corresponding experimental list started. At the end of the experiment subjects were requested to provide their age, gender and handedness.

### Results

Subjects who failed to retype at least 36 of the 48 sentences were excluded from the analysis ($n = 14$). Individual trials were excluded if the lexical decision was incorrect, too slow (>3500ms) or too fast (<200 ms). Only subjects with at least 154 (80 %) correct lexical decisions were included in the analysis ($n = 1$ subject excluded). Trials in which the reading time for the sentence was too short (<350ms) were excluded. Lexical decision RTs deviating more than 2.5 standard deviations from the mean of the corresponding condition (Polarity x Relation x Subject) were also excluded. Subjects who were left with less than eight data points per condition after the cleaning procedure were eliminated ($n = 4$). 18,37% of the initial subjects’ datasets was excluded.

The data were analyzed with linear mixed effect models using the *lme4* package in *R* (Bates, 2005). In order to run a sanity check and ensure that both plausible and implausible alternatives were activated more strongly than unrelated, Model 1 was fit to the data *unrelated* trials

1 $$\begin{aligned} rt \sim Relation + (1 |Item) + (1 |Subject) \end{aligned}$$

and compared against the baseline Model 2

2 $$\begin{aligned} rt \sim 1 + (1 |Item) + (1 |Subject) \end{aligned}$$

through a Likelihood-Ratio Test (LRT). Treatment coding was employed, with *unrelated* as reference level. Model 1 explained the data significantly better than Model 2 ($\chi ^2(2) = 102.56, p <.001$). Both the plausible and implausible alternatives differed significantly from the unrelated words ($\beta = -18.49$, $p <.001$ and $\beta = -11.25$, $p <.001$ respectively). On the other hand, a model with Polarity as fixed effect did not provide any improvement over Model 2 (${\chi }^2(1) = 0.21$, $p =.65$).

In order to test for the interaction of Relation and Polarity, unrelated words were omitted. A null model (Model 3), which included the two fixed factors Relation and Polarity, as well as items and subjects as random factors, was compared to Model 4, additionally including the interaction effect. Default treatment coding was employed, with *aff:plausible alternative* as reference level. These models and this analysis were preregistered after they were employed on the pilot data (https://osf.io/7qxne?mode= &revisionId= &view_only=) and are then used across experiments for consistency.^2^

3 $$\begin{aligned} rt \sim Relation + Polarity + (1 |Item) + (1 |Subject) \end{aligned}$$

4 $$\begin{aligned} rt \sim Relation * Polarity + (1 |Item) + (1 |Subject) \end{aligned}$$

**Fig. 1:**
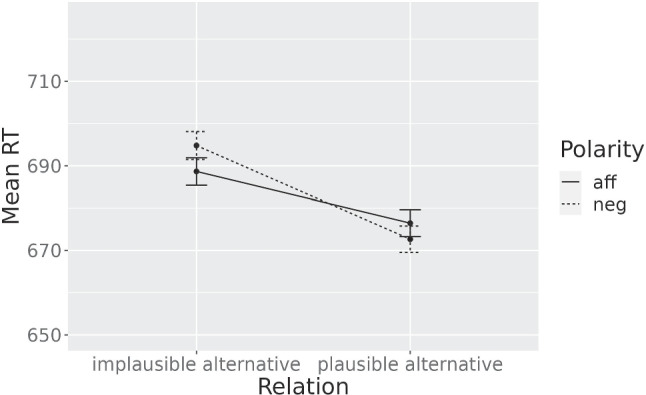
**Experiment**1 - Mean RTs. Error bars represent ± se of the means

Models 3 and 4 were fit to the experimental data. The interaction just missed the significance level (${\chi }^2(1) = 3.24$, $p =.07$). The mean RTs per condition are shown in Fig. 1. Model 4’s estimates for the fixed effects across all the experiments reported in this paper can be found in Table 1.

**Table 1:** **Model 4.** Fixed effects estimates

	Experiment 1	Experiment 2
(Intercept)	656.45	679.68
Polarityneg	0.52	0.33
Relationimplausible alternative	16.36	23.66
Polarityneg:Relationimplausible alternative	16.90	12.62

The pattern of means consistently adheres to the hypothesis, and the interaction just misses the significance level. A consideration to make is that not all plausible alternatives were *equally good* alternatives: some had higher cloze frequencies than others, and for 19 items cloze frequencies are not available as they were crafted by intuition. We can expect the plausible alternatives with the highest cloze frequencies to be more likely candidates for an enhanced activation, as a larger proportion of subjects can be expected to prefer them. This possibility was explored in a post hoc analysis.

## Post hoc analysis

In order to explore the role of alternative *goodness*, we analysed the data of Experiment 1 whilst retaining only the items with a cloze frequency above the median (>28).

**Fig. 2:**
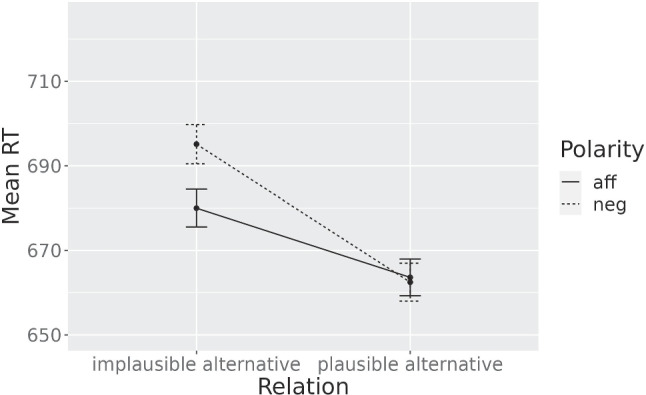
**Post hoc Analysis** - Mean RTs when retaining only the items with the highest cloze frequencies. Error bars represent ± se of the means

A main effect of Relation showed ($\chi ^2(2) = 68.64, p <.001$): plausible and implausible alternatives differed significantly from the unrelated words ($\beta = -32.58$, $p <.001$ and $\beta = -9.00$, $p <.05$ respectively). No effect of Polarity was observed (${\chi }^2(1) = 2.36$, $p =.12$), but there was a significant Relation x Polarity interaction (${\chi }^2(1) = 4.23$, $p <.05$) in the expected direction: plausible alternatives were facilitated after negation with respect to implausible alternatives, compared to the pattern of activation after affirmatives. The *lmerTest* ANOVA table for the interaction model is in the Appendix (Table 4). The mean RTs per condition are shown in Fig. 2. In conclusion, the goodness of alternatives seems to be an influential factor driving the effect.^3^

## Experiment 2

Based on the results of the post hoc analysis of Experiment 1 on the *best* items, we ran a second Experiment to investigate the expected interaction on a new sample. For that, we first conducted a power analysis on Experiment 1 selecting only the 48 best items in terms of the cloze frequencies obtained in our cloze task. The power analysis with 1000 simulations ($\alpha =.05$) resulted in 85.8% power when employing 540 subjects.

### Participants

Data was collected until 540 usable participants were reached. In total, 577 subjects were tested (382 female, 184 male, 11 diverse) with an age range of 18 to 59 years ($mean=26.40$, $sd=8.48$). Subjects who stated that they were not native German speakers ($n = 4$), as well as subjects who stated to have already participated in a very similar study (e.g. Experiment 1 or a previous version) ($n = 20$) were excluded from the analysis. The recruitment took place via Prolific (Palan and Schitter, 2018). All subjects gave written informed consent.

### Materials

We used the 48 items of Experiment 1 (i.e. half) with the highest cloze frequencies. Fillers ($n=48$) and target words were created analogously to the previous experiments. The mean length for the *plausible alternatives* was 6.33 ($sd=2.39$), 6.31 ($sd= 1.84$) for the *implausible alternatives* and 6.27 ($sd= 2.03$) for the *unrelated* targets. Plausible alternatives did not differ from implausible alternatives ($t(47) = 0.06$, $p =.95$), nor from unrelated targets ($t(47) = 0.14$, $p =.89$). Implausible alternatives and unrelated targets were also paired ($t(47) = 0.10$, $p =.92$). The mean frequency for the alternatives was 29047 ($sd=75968$), 24070 ($sd=39998$) for related and 42752 ($sd=132151$) for unrelated targets. Again, all the pairs of conditions were matched: plausible and implausible alternatives ($t(47)=0.40$, $p=.69$), plausible alternatives and unrelated ($t(47)=-0.62$, $p=.54$), implausible alternatives and unrelated ($t(47)=-0.92$, $p=.36$). Plausible alternatives were significantly more similar to the noun ($mean=0.70$, $sd=0.18$) not only with respect to unrelated targets ($mean=0.21$, $sd=0.15$; $t(47)=14$, $p<.001$), but also to implausible alternatives ($mean=0.47$, $sd=0.20$; $t(47)=5.8$, $p<.001$). Implausible alternatives were more similar to the noun than unrelated targets ($t(47)=9.2$, $p<.001$).

### Procedure

The procedure was the same as in Experiment 1.

### Results

The data analysis procedure was the same as in Experiment 1. Nine subjects failed to retype at least 18 of the 24 to-re-type sentences. Three subjects did not satisfy the minimum 80% accuracy criterion in the lexical decision task. Finally, four subjects were left with less than four observations in at least one experimental condition and were therefore excluded. In total, 6,64% of the collected subjects’ datasets was excluded.

**Fig. 3:**
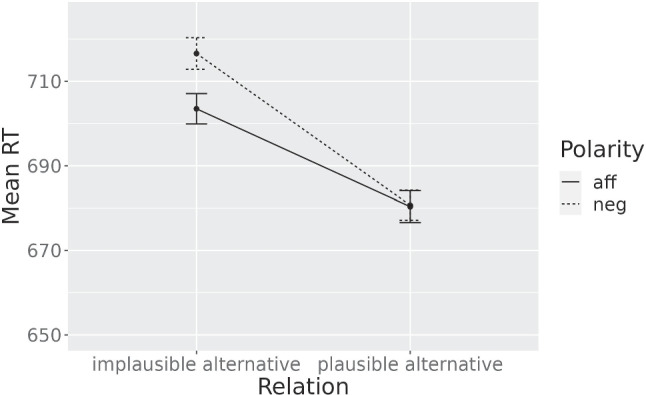
**Experiment**2 - Mean RTs when retaining only the items with the highest cloze frequencies. Error bars represent ±se of the means

Model 1 explained the data significantly better than Model 2 ($\chi ^2(2) = 126.49, p <.001$). In contrast to the prior experiment, the plausible alternatives - but not the implausible alternatives - differed significantly from the unrelated words ($\beta = -32.45$, $p <.001$ and $\beta = -2.67$, $p=.41$, respectively). This is maybe attributable to the fact that the best items that we selected for Experiment 2 by chance have particularly high mean frequency in the unrelated condition. Although the t-test shows no significant difference in the means, this might be driven by the larger standard deviations and reduced degrees of freedom. As in the prior experiment, a model with Polarity as fixed effect did not provide any improvement over Model 2 (${\chi }^2(1) = 1.20$, $p =.27$). Models 3 and 4 were again fit to the experimental data. This time, the interaction reached the significance level (${\chi }^2(1) =$ 3.87, $p <.05$). There was a significant Relation x Polarity interaction in the expected direction with plausible alternatives being more facilitated after negation with respect to implausible alternatives, compared to the pattern of activation after affirmatives. The mean RTs per condition are shown in Fig. 3,^4^

## Internal meta-analysis

**Fig. 4:**
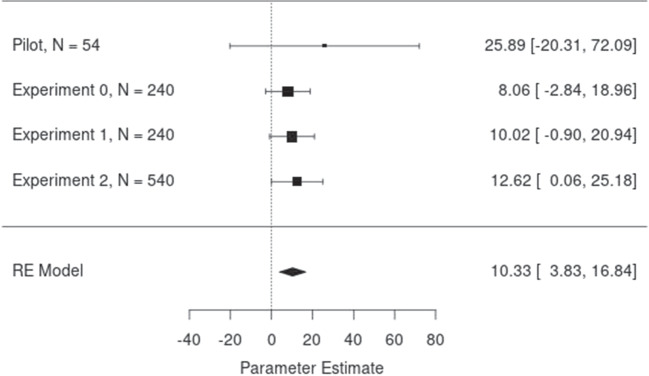
**Meta-Analysis** - Forest plot of the meta-analysis on the interaction effect of Polarity and Prime-Target Relation

We ran a random-effects meta-analysis with the R package *metafor* (Viechtbauer, 2010) to determine the reliability of our effect across experiments, including the pilot and the discarded study (“Experiment 0”). The parameter coefficient for the interaction of Polarity and Prime-Target Relation is significant ($\beta = 10.33$, $95\% CI [3.83, 16.84]$, $se = 3.32$, $p <.01$). Figure 4 shows the forest plot of the meta-analysis.

## Discussion and conclusions

### Overview of the study

In natural language, negation often serves the purpose of correcting a previously held assumption. In this sense, it might prompt a search for a plausibly *correct* alternative. A correction implies some inaccuracy that is more justified, the more it is confusable with the rectification ($\approx$ the more it bears resemblance to it). In fact, it was shown that, out of context, plausible alternatives to negated nouns tend to be very similar to the noun, often exceeding the similarity between nouns connected by *and* within an affirmative sentence.

The current study was designed to investigate whether negation facilitates the activation of plausible alternatives with respect to affirmation. It has been demonstrated that the comprehension of linguistic constructions that are semantically represented through alternatives sets - such as sentences involving focused items - are reflected in actual psychological mechanisms of activation of contextually plausible alternatives (Braun and Tagliapietra, 2010; Yan and Calhoun, 2019; Husband and Ferreira, 2016). Negation processing, instead, has been only generally linked to inhibitory mechanisms (Beltrán et al., 2019, 2021; de Vega et al., 2016; Liu et al., 2020). Nonetheless, its corrective connotation gives reason to question whether it equally generates a search for plausible alternatives. For this sake, we designed a priming experiment where minimalistic sentences varying in polarity (affirmative vs. negative) were used as primes, and nouns varying in their relationship with respect to the noun in the sentence (plausible vs. implausible alternative) were used as targets. An interaction effect of Sentence Polarity and Prime-Target Relation was expected between plausible and implausible alternatives, with negation facilitating the activation of plausible alternatives.

Our first experiment was based on a previous version that employed item materials of the form *This is [a/an]/[not a/not an] X* (German *Das ist [ein/eine]/[kein/keine] X*). Using those sentential contexts, we had run a pilot study ($n=60$) which did not provide evidence for the effect, but displayed a numeric tendency in the expected direction. Based on a power analysis of the pilot study, we collected data from 240 usable subjects. The item materials were identical to those used in Experiment 1, except for the different sentential context. This larger experiment replicated the results of the pilot (no interaction, but overall means numerically in line with the expectations). Alongside, Capuano et al. (2021) determined that the sentential contexts of the form *This is (no) X* are particularly infelicitous in demonstrating differences between affirmative and negative sentences. Specifically, we showed consistently no significant difference between affirmative sentences of the form *This is a goat, and that is a ________* and negative sentences of the form *This is not a goat, it is a ________* in terms of the similarity of the given noun to the noun they tend to be completed with. The same study though did find such a difference for three other sentential contexts (e.g. *There is no goat here, but there is a ________ there*). We concluded that the *This* sentential contexts might be a case where the affirmative version conveys a corrective reading just like the negative version, leading to the production of a substitute state of affairs. These preliminary studies were therefore discarded, but we still employed them to establish a reasonable sample size for Experiment 1, which was aimed at testing a sentential context that in Capuano et al. (2021) had displayed a significantly different behaviour depending on polarity (i.e. the *There* context). Whereas the interaction just misses the significance level ($p =.07$), the pattern of results of Experiment 1 is consistent with the hypothesis.

The question arose, whether the non-significant effect is due to non-unequivocal activation preferences. Differently from contradictory predicates, non-contradictory items can give rise to differential distributions of alternatives’ activation. One can therefore expect some items to more uniformly give rise to the activation of a specific alternative across participants, i.e. the alternatives with the highest cloze probabilities. The plausible alternatives employed in our study differed in terms of cloze probability. In fact, an analysis retaining only the items with the highest cloze frequency alternatives (i.e. the *best* items) resulted in a significant interaction for Experiment 1. To collect additional evidence that the *goodness* of alternatives is the critical factor for the effect to emerge, we ran Experiment 2, based on a power analysis on the data for the *best* items of Experiment 1. Indeed, Experiment 2 produced the expected interaction, with the facilitation of *good* alternatives after negation.

### Discussion of the results

#### Interaction effect of polarity and prime-target relation

Our results show that, when employing properly powered designs and appropriate items, a facilitative effect of negation to plausible alternatives can be detected, thereby confirming our hypothesis that negation can activate plausible alternatives also in the case of non-binary predicates.

As already clarified in the introduction, this facilitation is not to be understood in absolute terms, but rather with respect to what happens in the baseline condition (implausible alternatives). Even though at first sight the means suggest that negation inhibits implausible alternatives rather than facilitates plausible alternatives (because negation shows slower RTs than affirmation for implausible alternatives, but not for plausible alternatives), we should not forget that negation is commonly associated with longer processing times. A direct comparison of Polarity levels within each Prime-Target Relation level is therefore not very informative to our main hypothesis: the slower RTs in the case of implausible alternatives might result from a general slowing effect of negation, and not from a specific inhibition of implausible alternatives.

#### Main effect of polarity

Contrary to our expectations, a main effect of sentence Polarity was never found. This could be due to our analysis being carried out exclusively on the reaction times of the lexical decision task. A slowing effect of negation might be visible only on sentence reading times - which were not analysed - without carrying over to the lexical decision task. Subjects determined themselves whether they were done reading the sentences before proceeding to the lexical decision task: by then, sentence processing might have been completed for both negative and affirmative sentences. Another possibility to consider is that precisely a facilitative effect of negation in the case of plausible alternatives might have wiped out a general Polarity effect.

### Future directions

The finding of an interaction is particularly meaningful when we consider that our experimental items suffered from shortcomings due to the difficulty to control for multiple sources of variance. The minimalistic contexts, employed for comparability with Capuano et al. (2021) and for ease of collection of the alternatives, might have led the subjects to focus only on the noun and on the presence of the negation marker instead of reading the whole sentence. This might have rendered our paradigm more akin to a single word priming paradigm. In fact, the control task to retype the prime sentence did not ensure sentence-level comprehension but a more *semantic* task was difficult to devise with such minimalistic sentences. Both points potentially hindered the detection of the effect, which as a consequence becomes even more outstanding. Noticeably, the direction of the interaction in terms of trends remains consistent across all experiments, which makes it less likely that the findings are due to chance. This is further confirmed by the meta-analysis, which registers a significant overall interaction. Nevertheless, further effort should go into developing an alternative experimental design that can more neatly isolate the effect.

The use of minimalistic contexts also puts some limitations to the generalisability of the conclusions. Future investigation will need to extend the findings to other sentential contexts to make sure they are applicable to a general use of negation. The minimalistic contexts also circumscribed the types of alternatives investigated. In our study, plausible and implausible alternatives, apart from being distinguishable through differences in cloze task probabilities, stand systematically in different semantic relations with the prime noun: plausible alternatives are cohyponyms, whereas implausible alternatives stand in different relations to the prime. Similarly, Husband and Ferreira (2016)’s *contrastive associates* seem to be cohyponyms, whereas *non-contrastive associates* are associates in other kinds of semantic relations. The same goes for Yan and Calhoun (2019)’s materials. Braun and Tagliapietra (2010) go even further and sometimes employ different parts of speech such as advjectives as non-contrastive alternatives. We think that good alternatives are not limited to the relationship of cohyponymy, but by availability and how much overlap there is between the entities that is functional to the substitution in a specific context. Cohyponyms can substitute an entity in a wide range of contexts, so they tend to be the best alternatives when the contexts are not too restrictive. If negation activates a general search for plausible alternatives that are *contextually relevant*, we should be able to detect the same effect independently of semantic relation.

Finally, although we cannot exclude that the interaction effect might be driven by an inhibition in the activation of implausible alternatives rather than by the enhanced activation of plausible ones, Dennison and Schafer (2017) provides evidence in line with a progressive deactivation of less relevant alternatives, both in the case of contrast expressed through intonational form and in the case of contrast expressed through explicit negation. The study though is again confined to binary predicates. Further research in the time course of these activations after non-binary negation is needed to set apart the two processes more clearly (inhibition vs. enhanced activation).

**Table 2:** **Experiment**1 - ANOVA table provided by the *lmerTest* package for the same analyses as in the main text

	Sum Sq	Mean Sq	NumDF	DenDF	F value	Pr(>F)
Polarity	5370.65	5370.65	1.00	14139.72	0.19	0.6614
Relation	1095450.78	1095450.78	1.00	14144.51	39.13	0.0000
Polarity:Relation	90776.33	90776.33	1.00	14140.10	3.24	0.0718

$$rt \sim Polarity * Relation + (1|Item) + (1|Subject)$$

**Table 3:** **Experiment**1 - Maximal model reduced until convergence

	Sum Sq	Mean Sq	NumDF	DenDF	F value	Pr(>F)
Polarity	5065.38	5065.38	1.00	235.79	0.19	0.6664
Relation	221686.98	221686.98	1.00	92.33	8.15	0.0053
Polarity:Relation	93512.24	93512.24	1.00	13822.81	3.44	0.0637

$$rt \sim Polarity * Relation + (1 + Relation|Item) + (1 + Polarity|Subject)$$

### Conclusive remarks

*Alternatives* in the context of negation most commonly refer to the contrast between the negated and the expressed proposition (Repp and Spalek, 2021); particular attention had been devoted to the time course of their access and integration in the mental model of the listener. Alternatives as the ones we refer to in the present study are traditionally investigated under the heading of *focus alternatives*, especially in relation to prosodically and syntactically marked focus. Different types of alternatives interact though, and disparate domains of alternatives have also been investigated jointly (Repp and Spalek, 2021). The current study suggests that negation functions as a (contrastive) focus marker, triggering focus alternatives without discourse context, and without explicit prosodic marking. In fact, the pragmatic functions attributed to negation resonate with the notion of contrast delineated by Zimmermann et al. (2008): contrastive focus expresses the speaker’s assumption that the listener does not expect the upcoming information; as such, it signals the need for a shift in the interlocutor’s assumptions and an update of their common ground. Therefore, relevant alternatives are not simply dictated by semantic similarity, but by speakers’ expectations on the status of their common ground, whereby semantic similarity is just a byproduct of the presentation of stimuli out of context. The discourse context is responsible for the restriction and therefore selection of the relevant alternatives.

Orenes et al. (2014) showed that, after hearing a sentence such as *The figure is not green*, subjects ended up fixating the alternative (e.g. a blue figure) whenever only two concurrent alternatives were offered by a visual world (a green and a blue figure), but stayed fixated on the green figure when more alternatives were presented (e.g. green, blue, yellow, pink). They conclude that alternatives are activated when there are only two, but not when there are more than two. Whereas no generalized experience suggests that *blue* is a better alternative to *green* than *yellow* though, there is reason to assume that this is not always the case whenever more than one alternative is available (e.g. some entities can be widely agreed upon to be *better alternatives* than others). As a more general criterion, our study suggests that the prominence (rather than the number) of potential alternatives might be the decisive factor determining the activation.

In conclusion, the evidence presented in this study supports the hypothesis that negation involves processing mechanisms that favour plausible alternatives. This seems the case even for non-binary negation, but might be confined to instances where the negated content displays particularly prominent alternatives. The psychological relevance of alternatives in the processing of negation is akin to the mechanisms demonstrated to be at play in the comprehension of structures marked with phonological focus. Therefore, the notion of contrastive focus might need to broaden to include negative constructions. The nature of the ‘preference’ for plausible alternatives in the case of negation is still unclear, potentially corresponding either to a preferential activation of plausible alternatives or to a selective deactivation of implausible ones. It is possible that the evidence for the use of inhibitory mechanisms in negation comprehension reflects exactly the process of deactivation of implausible alternatives. This issue needs further investigation and might benefit from the inspection of the time course of the candidates’ activation.

## Data Availability

The materials, the data and the analyses scripts of all the experiments presented in this paper can be found at https://osf.io/p5g8u/?view_only=3f40dc06bfa84ac09ce66120c6e71ae4.

## Appendix

### Additional analyses

**Table 4:** **Post hoc Analysis** - Interaction model fit on the best items

	Sum Sq	Mean Sq	NumDF	DenDF	F value	Pr(>F)
Polarity	90600.99	90600.99	1.00	6868.14	3.31	0.0690
Relation	1047570.66	1047570.66	1.00	6949.56	38.23	0.0000
Polarity:Relation	115858.85	115858.85	1.00	6866.61	4.23	0.0398

$$rt \sim Polarity * Relation + (1|Item) + (1|Subject)$$

**Table 5:** **Experiment**1 - Maximal model reduced until convergence with cloze probability as covariate

	Sum Sq	Mean Sq	NumDF	DenDF	F value	Pr(>F)
Polarity	85387.90	85387.90	1.00	2874.09	3.21	0.0735
Relation	35606.72	35606.72	1.00	75.64	1.34	0.2513
Cloze.Percentage	28678.23	28678.23	1.00	75.26	1.08	0.3028
Polarity:Relation	325.22	325.22	1.00	11242.33	0.01	0.9120
Polarity:Cloze.Percentage	139690.98	139690.98	1.00	11202.68	5.24	0.0221
Relation:Cloze.Percentage	188626.83	188626.83	1.00	76.06	7.08	0.0095
Polarity:Relation:Cloze.Percentage	22598.68	22598.68	1.00	11170.36	0.85	0.3571

$$rt \sim Polarity * Relation * Cloze.Percentage + (1 + Relation|Item) + (1 + Polarity|Subject)$$

**Table 6:** **Experiment**2 - ANOVA table provided by the *lmerTest* package for the same analyses as in the main text

	Sum Sq	Mean Sq	NumDF	DenDF	F value	Pr(>F)
Polarity	185606.04	185606.04	1.00	16295.49	4.28	0.0385
Relation	3787805.83	3787805.83	1.00	16296.77	87.37	0.0000
Polarity:Relation	167847.98	167847.98	1.00	16295.55	3.87	0.0491

$$rt \sim Polarity * Relation + (1|Item) + (1|Subject)$$

**Table 7:** **Experiment**2 - Maximal model reduced until convergence

	Sum Sq	Mean Sq	NumDF	DenDF	F value	Pr(>F)
Polarity	182975.71	182975.71	1.00	15734.07	4.28	0.0386
Relation	818165.24	818165.24	1.00	46.37	19.14	0.0001
Polarity:Relation	165304.56	165304.56	1.00	15731.84	3.87	0.0493

$$rt \sim Polarity * Relation + (1 + Relation|Item) + (1 + Relation|Subject)$$

**Table 8:** **Experiment**2 - Maximal model reduced until convergence with cloze probability as covariate

	Sum Sq	Mean Sq	NumDF	DenDF	F value	Pr(>F)
Polarity	9836.90	9836.90	1.00	15830.88	0.23	0.6314
Relation	11251.58	11251.58	1.00	44.65	0.26	0.6104
Cloze.Percentage	3012.13	3012.13	1.00	45.68	0.07	0.7918
Polarity:Relation	62.25	62.25	1.00	15930.69	0.00	0.9696
Polarity:Cloze.Percentage	914.51	914.51	1.00	15642.45	0.02	0.8837
Relation:Cloze.Percentage	170715.08	170715.08	1.00	44.50	3.99	0.0518
Polarity:Relation:Cloze.Percentage	17406.98	17406.98	1.00	15946.44	0.41	0.5234

$$rt \sim Polarity * Relation * Cloze.Percentage + (1 + Relation|Item) + (1 + Relation|Subject)$$

## Footnotes

1 For simplicity and for the sake of the procedure explanation, material is translated to English.

2 As LRT was shown to produce anti-conservative *p* values, especially with few observations (Kuznetsova et al., 2017), we also report the results of the analysis with the *lmerTest* outputs in the Appendix (Table 2), which do not diverge at all from our main analysis. As requested by a reviewer, we also report the results of the maximal models that we could fit for each experiment without encountering convergence issues. See Table 3 for the results of the maximal model fit on Experiment 1. Similarly, these do not diverge from the main analysis.

3 As requested by a reviewer, we additionally ran an analysis with Cloze Percentage as a covariate. The model is in Table 5 in the Appendix. In that model the three-way interaction with Cloze Percentage is not significant, but this does not invalidate our exploratory analysis, because the goodness of alternatives might not be a strictly continuous measure. Moreover, the intuition of the post hoc analysis was tested in a completely new experiment, erasing the possibility of any posthoc manipulation.

4 The analyses with *lmerTest* with the maximal converging model and the model with the cloze probabilities are in the Appendix (Tables 6, 7 and 8). The results do not diverge from the main analyses nor from the considerations made on Experiment 1, except for the main effect Polarity, which reaches significance with *lmerTest*.
